# Hydrothermal Synthesis of MoS_2_/SnS_2_ Photocatalysts with Heterogeneous Structures Enhances Photocatalytic Activity

**DOI:** 10.3390/ma16124436

**Published:** 2023-06-16

**Authors:** Guansheng Ma, Zhigang Pan, Yunfei Liu, Yinong Lu, Yaqiu Tao

**Affiliations:** 1 College of Materials Science and Engineering, Nanjing Tech University, Nanjing 211800, China; 202061203287@njtech.edu.cn (G.M.); panzhigang@njtech.edu.cn (Z.P.); yfliu@njtech.edu.cn (Y.L.); yinonglu@njtech.edu.cn (Y.L.); 2 State Key Laboratory of Materials-Oriented Chemical Engineering, Nanjing 211800, China

**Keywords:** MoS_2_, SnS_2_, photocatalysis, composite catalyst, visible light degradation

## Abstract

The use of solar photocatalysts to degrade organic pollutants is not only the most promising and efficient strategy to solve pollution problems today but also helps to alleviate the energy crisis. In this work, MoS_2_/SnS_2_ heterogeneous structure catalysts were prepared by a facile hydrothermal method, and the microstructures and morphologies of these catalysts were investigated using XRD, SEM, TEM, BET, XPS and EIS. Eventually, the optimal synthesis conditions of the catalysts were obtained as 180 °C for 14 h, with the molar ratio of molybdenum to tin atoms being 2:1 and the acidity and alkalinity of the solution adjusted by hydrochloric acid. TEM images of the composite catalysts synthesized under these conditions clearly show that the lamellar SnS_2_ grows on the surface of MoS_2_ at a smaller size; high-resolution TEM images show lattice stripe distances of 0.68 nm and 0.30 nm for the (002) plane of MoS_2_ and the (100) plane of SnS_2_, respectively. Thus, in terms of microstructure, it is confirmed that the MoS_2_ and SnS_2_ in the composite catalyst form a tight heterogeneous structure. The degradation efficiency of the best composite catalyst for methylene blue (MB) was 83.0%, which was 8.3 times higher than that of pure MoS_2_ and 16.6 times higher than that of pure SnS_2_. After four cycles, the degradation efficiency of the catalyst was 74.7%, indicating a relatively stable catalytic performance. The increase in activity could be attributed to the improved visible light absorption, the increase in active sites introduced at the exposed edges of MoS_2_ nanoparticles and the construction of heterojunctions opening up photogenerated carrier transfer pathways and effective charge separation and transfer. This unique heterostructure photocatalyst not only has excellent photocatalytic performance but also has good cycling stability, which provides a simple, convenient and low-cost method for the photocatalytic degradation of organic pollutants.

## 1. Introduction

Today’s industrialized and urbanized world is facing severe energy shortages and environmental pollution problems. The excessive use of organic dye and the indiscriminate release of organic pollutants cause serious damage to ecosystems and have serious effects on future generations [1]. Moreover, the organic ingredients in our living environment are difficult to degrade and toxic in nature. There have been many methods to solve the problem of organic dye contamination in environment, such as adsorption [2,3], membrane separation [4,5], biological decomposition [6], chemical oxidation [7], electrocatalysis [8] and photocatalysis [9,10] decomposition. Among them, the use of solar photocatalysis for the degradation of organic pollutants is considered one of the most promising and efficient strategies [11,12].

Transition metal sulfides have attracted a lot of attention in wastewater treatment because of their high specific surface area, high surface activity and special microstructure. In recent years, MoS_2_ has been widely used in organic dye decomposition due to its low cost, high abundance and noble-metal-like activities [13,14,15]. MoS_2_ has a graphene-like layered structure with three crystal phases: 1T, 2H and 3R [16]. In a natural state, MoS_2_ is usually present in the steady 2H phase, which exhibits semiconducting properties [17,18]. However, the low density of active sites and relatively poor conductivity of 2H-MoS_2_ lead to limited photocatalytic activity [19,20]. Compared with 2H-MoS_2_, the metallic 1T-MoS_2_ phase has the advantages of significant conductivity and a high density of marginal active sites at room temperature and shows better performance in photocatalysis [21,22,23,24]. So far, most 1T-MoS_2_ is fabricated as two-dimensional nanosheets to construct hybrid structures with nanoconjunctions [25,26,27].

Li et al. [28] prepared two-dimensional heterostructured MoS_2_/g-C_3_N_4_ (graphite-C_3_N_4_) photocatalysts using a facile impregnation–calcination method. The experimental results showed that surface MoS_2_ nanosheets were successfully loaded horizontally onto g-C_3_N_4_ nanosheets. Meanwhile, the two-dimensional heterojunction formed between g-C_3_N_4_ nanosheets and MoS_2_ nanosheets improved the separation efficiency and charge transfer rate of photogenerated electrons. One of the synthesized samples, MCNNs-3 (3 wt% MoS_2_ in MoS_2_/g-C_3_N_4_ heterojunction), with a catalyst content of 0.8 g/L, reduced the concentration of rhodamine B (RhB) by about 96% after 20 min of irradiation. Chen et al. [29] prepared MoS_2_/TaON (tantalum oxynitride) hybrid nanostructures by a hydrothermal method. This work showed that the photocatalytic degradation of rhodamine B (RhB) on Ta1Mo1 (mass ratio of TaON:MoS_2_ = 1:1) was about 65% after 2 h of visible light irradiation, which was about five times higher than that of pure TaON. In addition, MoS_2_/SiO_2_/TaON ternary photocatalysts were constructed to further improve the photocatalytic performance. When the mass ratio of Ta8Si1 (TaON:SiO_2_ = 8:1) to MoS_2_ was 1:1, the degradation rate of RhB reached 75% under 2 h of visible light irradiation. Yin et al. [30] synthesized two kinds of MoS_2_ and PbBiO_2_Cl nanosheets by the solvothermal method and then prepared a novel 2D/2D MoS_2_/PbBiO_2_Cl photocatalyst by mechanical stirring at room temperature. The resulting experiments showed that 1 wt% of MoS_2_/PbBiO_2_Cl showed stronger photocatalytic performance and 80% of rhodamine B (RhB) could be completely degraded within 120 min, whereas the photocatalytic activity decreased when the content of MoS_2_ was higher.

Composites containing MoS_2_ with other similar materials are an effective way to enhance the photocatalytic ability of the material. SnS_2_ has a narrow band gap of 2.0 to 2.3 eV and is a low-cost, non-toxic CdI_2_-type layered semiconductor [31,32,33]. According to the literature [34,35,36], SnS_2_ is a relatively stable visible-light-driven photocatalyst in the degradation of organic compounds. However, like most semiconductor photocatalysts, SnS_2_ also has the disadvantage of high recombination rates of photogenerated electrons and holes, resulting in low photocatalytic efficiency [37]. Among the modification strategies explored to improve the photocatalytic efficiency of SnS_2_, the combination with a suitable semiconductor or other components (e.g., graphene) facilitates the separation of photogenerated electrons and holes through interfacial charge transfer [38,39,40]. Zhang et al. [41] prepared 2D/2D-type SnS_2_/g-C_3_N_4_ (graphite–C_3_N_4_) heterojunction photocatalysts using an ultrasonic dispersion method. The electron microscopic characterization analysis showed that a large contact zone was induced at the heterojunction interface due to the lamellar structure of both the SnS_2_ and g-C_3_N_4_ materials. In the photoluminescence spectra, it can also be shown that the photo-coordination effect of the SnS_2_/g-C_3_N_4_ heterojunction effectively enhances the interfacial carrier transfer, leading to enhanced charge separation during the photocatalytic reaction.

Due to the outstanding reactivity of both MoS_2_ and SnS_2_ in the photocatalytic degradation of organic dyes, the structures of MoS_2_ and SnS_2_ were coupled to construct a heterogeneous structure to enhance the degradation of organic dyes. Compared with previous work, this experiment is further improved: firstly, by changing the synthesis method of the sample and the synthesis conditions, the high temperature and high energy consumption in the experiment as well as the shortened reaction time are avoided; secondly, the synthesis steps are simpler and less cumbersome; and finally, the reactants are easily available throughout the experiment and the heterogeneous structure is binary, which can efficiently solve the cost problem in the application.

In this work, we used a convenient hydrothermal method to obtain MoS_2_/SnS_2_ composite catalysts with SnS_2_ nanosheets grown on MoS_2_ nanoparticles. By adjusting the hydrothermal time of the reaction (12 h, 14 h and 16 h) and changing the molar ratio of the substances (1:1, 2:1, 3:1 and 4:1 atomic molar ratio of molybdenum–tin), a heterogeneous structure was constructed between MoS_2_ and SnS_2_ after the hydrothermal reaction, resulting in a semiconducting composite photocatalyst with a narrow band gap. Theoretically, the narrowing of the band gap of the material can effectively improve the absorption of visible light and the catalyst material can produce a large number of electrons and holes when light can be irradiated. In addition, the heterostructure can effectively modulate the electronic structure of the complex system while promoting electron transport between the interfaces more effectively, thus improving the photocatalytic ability. This work highlights that the construction of heterojunctions between two substances may be an attractive method for the removal of pollutants from industrial wastewater.

## 2. Materials and Methods

### 2.1. Raw Materials

Thiourea (CH_4_N_2_S) was purchased from Shanghai Ling Feng Chemical Reagent Co. (Shanghai, China). Tin chloride pentahydrate (SnCl_4_·5H_2_O, 99%) was supplied by Shanghai Test Four Hervey Chemical Co. (Shanghai, China). Ammonium molybdate tetrahydrate ((NH_4_)_6_Mo_7_O_24_·4H_2_O, 99%) was purchased from Sinopharm Chemical Reagent Co. (Shanghai, China). Hydrochloric acid (HCl) was supplied by Yonghua Chemical Co. (Changshu, China). Methylene blue (MB) was supplied by Tianjin Chemical Reagent Research Co. (Tianjin, China). Except for hydrochloric acid, which is superiorly pure, the rest of the chemical reagents are of analytical grade and were used without further purification.

### 2.2. Synthesis of Photocatalysts

**Figure 1:**
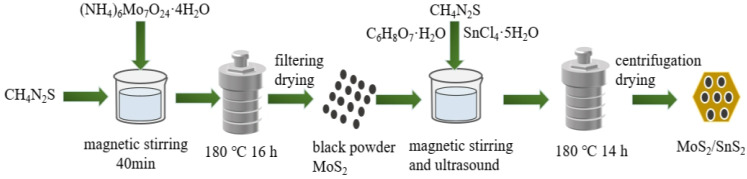
Schematic diagram of the synthesis procedure of MoS_2_/SnS_2_ catalysts.

The fabrication steps involved in the synthesis of the MoS_2_/SnS_2_ composite catalysts are schematically illustrated in Figure 1. First, the synthesis of MoS_2_ nanoparticles [42]: MoS_2_ nanoparticles were synthesized by the hydrothermal method. Typically, 1.0592 g of (NH_4_)_6_Mo_7_O_24_·4H_2_O and 1.828 g of CH_4_N_2_S were dissolved in 60 mL of deionized water at room temperature with continuous stirring until complete dissolution. The mixed solution was then transferred to a 100 mL Teflon-lined autoclave and kept at 180 °C for 16 h. Then, the obtained black precipitate was dried at 80 °C for 2 h.

Preparation of MoS_2_/SnS_2_ composite catalysts [43] with different reaction times (12 h,14 h and 16 h): the black MoS_2_ powder was weighed according to the ratio and dispersed in 60 mL deionized water to form a suspension. Certain proportions of SnCl_4_·5H_2_O and CH_4_N_2_S were added to the suspension; then, a certain amount of 1 mol/L hydrochloric acid was added to adjust the mixed solution to acidity. Finally, the mixture was transferred to a 100 mL Teflon-lined autoclave and kept at 180 °C for a certain time. After the reaction was completed, the catalyst was collected by centrifugation and dried at 80 °C for 2 h to obtain the catalyst. The catalysts were named MS12-2-H, MS14-2-H and MS16-2-H according to the reaction time and the atomic molar ratio of molybdenum to tin. 

Preparation of MoS_2_/SnS_2_ composite catalysts with different Mo/Sn atomic molar ratios (1:1, 2:1, 3:1 and 4:1): the reactants were weighed according to the ratios and the synthesis steps were the same as above; the final composite catalysts were named MS14-1-H, MS14-2-H, MS14-3-H and MS14-2-H according to the naming principle.

### 2.3. Structural Characterization

Powder X-ray diffraction patterns were obtained using a Rigaku Smart Lab diffractometer with Cu Kα (λ = 0.154178 nm) as the radiation source. The morphology of the samples was measured using a JSM-6510 scanning electron microscope. Nitrogen adsorption–desorption isotherms were measured at −196 °C using a specific surface area and pore-size analyzer, the V-Sorb 1800. The samples were all pretreated at 105 °C for 12 h prior to measurement. Electrochemical impedance was tested with a CH1660E electrochemical workstation. X-ray photoelectron spectra were obtained with a KRATOS AXIS SUPRA.

### 2.4. Photocatalytic Degradation and Photoelectrochemical Test

The catalytic performances of the composite catalysts were evaluated by their ability to degrade the target pollutant, MB, under visible light using a 300 W xenon lamp as a visible light source. In each test, 30 mg of catalyst was dispersed in 80 mL of MB solution (15 mg/L). The mixed solution was stirred in the dark for 30 min prior to the test to achieve an adsorption–desorption equilibrium between the catalyst and the solution. The 7 mL suspension was removed every 10 min under visible light and centrifuged at 3500 r/min for 5 min. The absorbance of the solution at different reaction times was measured by UV–visible spectrophotometer. 

The determination of the concentration of organic dyes can be described by the Beer–Lambert law, and the amount of light absorbed by the solution follows the Beer–Lambert law. The specific equation is as follows [44]:

A = εbc(1)

where A is the absorbance, ε is the light absorption coefficient, b is the solution thickness and c is the dye concentration solution at the time of sampling. According to the Beer–Lambert law, the relationship between dye concentration and absorbed light is linear.

The electrochemical impedance test was carried out in a CH1660E electrochemical workstation with a platinum electrode as the counter electrode and a saturated glycerol electrode as the reference electrode, and the corresponding open circuit voltage and frequency were set. In this experiment [43], catalyst-coated conductive glass was used as the working electrode, namely 20 mg of the catalyst dispersed into a mixture containing 40 uL of 5 wt% Nafion and 0.5 mL of anhydrous ethanol. After mixing well with ultrasound, 200 uL of the suspension was coated onto the surface of the conductive glass with a pipette gun; this was then dried naturally at room temperature. The working electrode for the electrochemical impedance was tested in 0.1 M Na_2_SO_4_ solution.

## 3. Results and Discussion

### 3.1. Characterization and Properties of Composite Catalysts Synthesized for Different Reaction Times

#### 3.1.1. XRD Characterization

**Figure 2:**
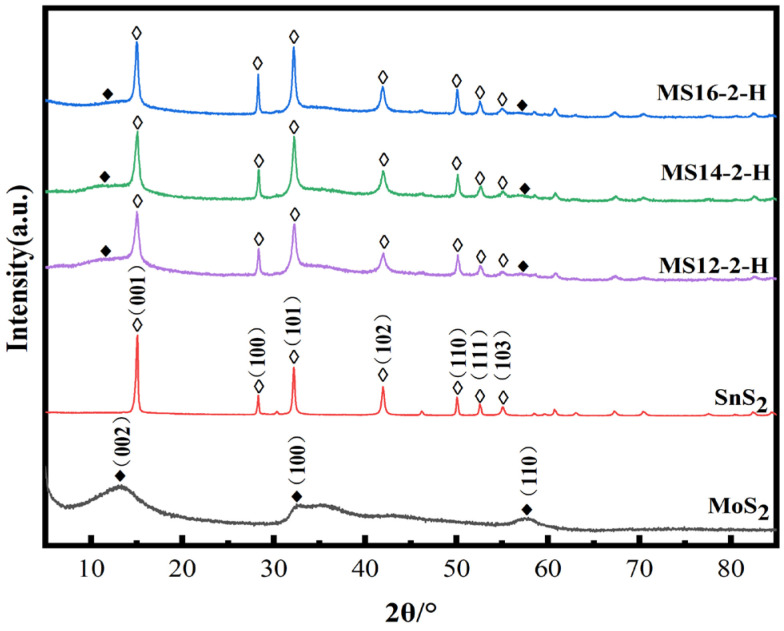
Powder X-ray diffraction patterns of MoS_2_/SnS_2_ composite catalysts with different reaction times.

Figure 2 represents the XRD patterns of the MoS_2_/SnS_2_ composite catalysts prepared from 1T-MoS_2_ synthesized by a hydrothermal reaction at 180 °C for different reaction times. It can be seen from the figure that the characteristic peaks of the SnS_2_ component at 2θ equal to 14.9°, 28.2°, 32.1°, 41.8°, 49.9°, 52.4° and 54.9° are clearly observed, which correspond to the (001), (100), (101), (102), (110), (111) and (103) SnS_2_ crystalline planes, respectively (JCPDS No. 23-0677) [45]. The SnS_2_ component is successfully synthesized in the MoS_2_/SnS_2_ composite catalysts. The characteristic peaks of MoS_2_ at 2θ equal to 10.9°, 32.8° and 57.2° are not clearly shown in the figure because of the unique lamellar structure and small grain size of MoS_2_ [46]. In addition, the layer spacing of MoS_2_ becomes larger during the reaction process, resulting in a shift of the (002) crystal plane to 10.9°. The presence of both MoS_2_ and SnS_2_ components in the synthesized catalysts without spurious peaks in the XRD patterns indicate the successful synthesis of MoS_2_/SnS_2_ composite catalysts.

With increasing reaction time, the characteristic peak of MoS_2_ gradually broadens and the characteristic peaks of SnS_2_ gradually narrow, indicating the stronger crystallinity of the SnS_2_ phase, which on the other hand also means that the structure of SnS_2_ in the reaction process is more complete.

#### 3.1.2. Morphology Analysis

**Figure 3:**
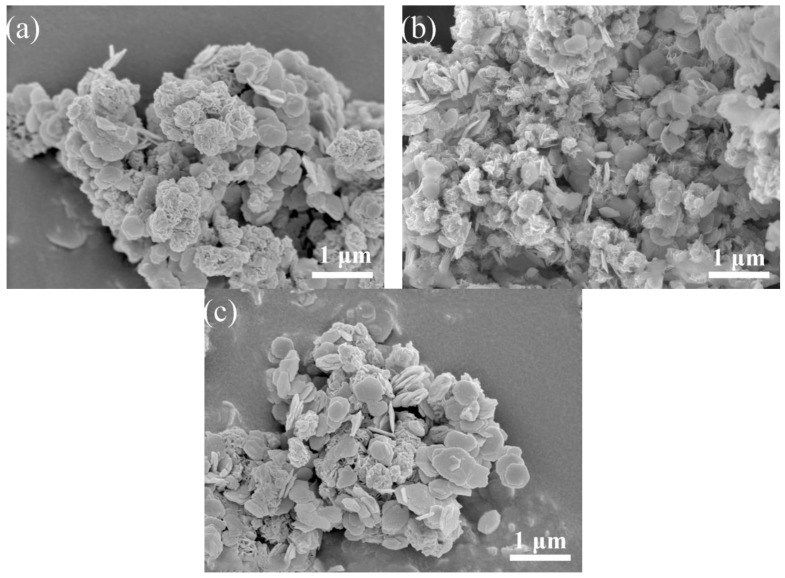
SEM images of MoS_2_/SnS_2_ composite catalysts: (**a**) MS12-2-H; (**b**) MS14-2-H; (**c**) MS16-2-H.

Scanning electron micrographs of the catalysts MS12-2-H, MS14-2-H and MS16-2-H synthesized at different times are shown in Figure 2. In Figure 3a, the hexagonal SnS_2_ nanosheets are grown on MoS_2_ nanosphere flowers [1], whereas the hexagonal SnS_2_ nanosheets are not uniformly distributed and a large portion of the nanoflake particles are not in contact with the nanosheets.

The morphology of the catalysts in Figure 3b changed considerably. The growth of SnS_2_ nanosheets on the surface of the flower-like morphology of MoS_2_ was not only more uniformly distributed but also the agglomeration of SnS_2_ nanosheets was slight, which was obviously different from the other catalysts and could expose more active sites. The SnS_2_ nanosheets in Figure 3c also have better crystallinity of the grains, although they are more uniformly distributed than in (a), which is consistent with the results for the XRD experiments.

#### 3.1.3. BET Measurements

Table 1 shows the results of the specific surface area test results. The nitrogen adsorption–desorption isotherms were measured at −196 °C after all the samples were pretreated at 105 °C for 12 h prior to measurement. The specific surface area of the composite catalyst showed a trend of increasing and then decreasing with the increase in the reaction time. The reaction time did not have a great influence on the average pore diameter in the range 2.32–2.34 nm and total pore volume of 0.003 cm^3^/g of the composite catalysts, which were almost negligible. Combined with the analysis of the SEM images of the composite catalysts, the specific surface area of the MoS_2_ and SnS_2_ materials was greatly enhanced due to the uniform growth of lamellar SnS_2_ particles on the surface of the flower-like MoS_2_ particles; thus, effectively improving the catalytic performance [47].

#### 3.1.4. Electrochemical Impedance Measurement

**Figure 4:**
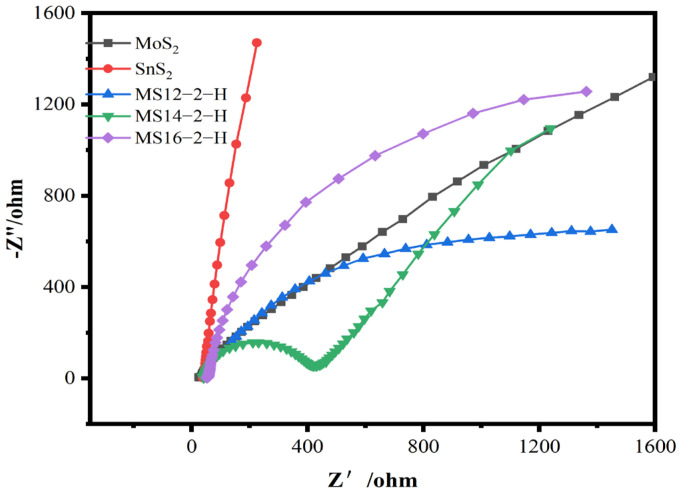
Electrochemical impedance diagram of MoS_2_/SnS_2_ composite catalysts at different reaction times.

Figure 4 shows the electrochemical impedance plots for the composite catalysts synthesized for different reaction times. In the electrochemical impedance diagram, the radius of the semicircle in the high-frequency region is positively correlated with the charge transfer resistance, reflecting the transfer characteristics of photogenerated electrons and holes in the catalysts under the light conditions. According to the test results, the impedance radius of the MoS_2_/SnS_2_ composite catalysts showed a trend of decreasing and then increasing as the reaction time increased, in which the MS14-2-H composite catalyst had the smallest impedance radius and presented a high charge transfer migration efficiency [48]. This also proves that a suitable reaction time has a great impact on the electronic structure of the two components in the catalysts, resulting in the improvement of their charge transfer capacity and thus the photocatalytic degradation performance.

#### 3.1.5. Photocatalytic Performance

**Figure 5:**
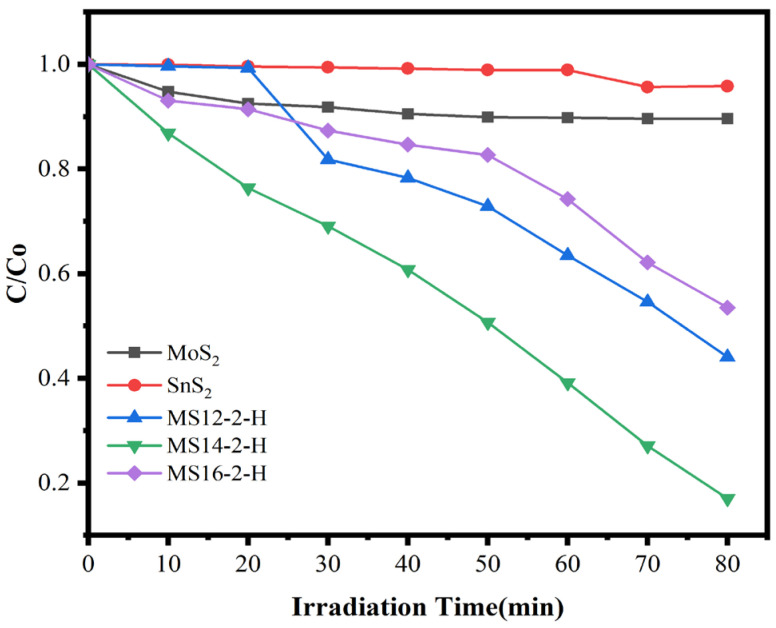
Degradation rate diagram of MoS_2_/SnS_2_ composite catalysts at different reaction times.

The degradation of methylene blue solution by MoS_2_/SnS_2_ composite catalysts formed at different reaction times is shown in Figure 5. From the figures, it can be observed that the composite catalyst degraded MB in visible light. Of the three catalysts, MS14-2-H exhibited the highest MB degradation rates. With the increasing of the synthesis time of the MoS_2_/SnS_2_ composite catalysts, a trend of enhancing and then weakening is observed, which is consistent with the test results of the electrochemical impedance of the composite catalysts. The length of the reaction time has an effect on the electronic structure of the synthesized composite catalyst, thus affecting the migration rate of photogenerated charges in visible light and producing an effect on the performance of the degradation of MB. From the degradation data, it was found that the best catalytic performance was achieved by sample MS14-2-H, which had an 83.0% degradation rate after 80 min of visible light irradiation. This was followed by sample MS12-2-H, which had a 55.9% degradation rate. This corroborates the previous results of the XRD, SEM and EIS analyses.

### 3.2. Characterization and Properties of Composite Catalysts in Different Mo–Sn Molar Ratios

#### 3.2.1. XRD Characterization

**Figure 6:**
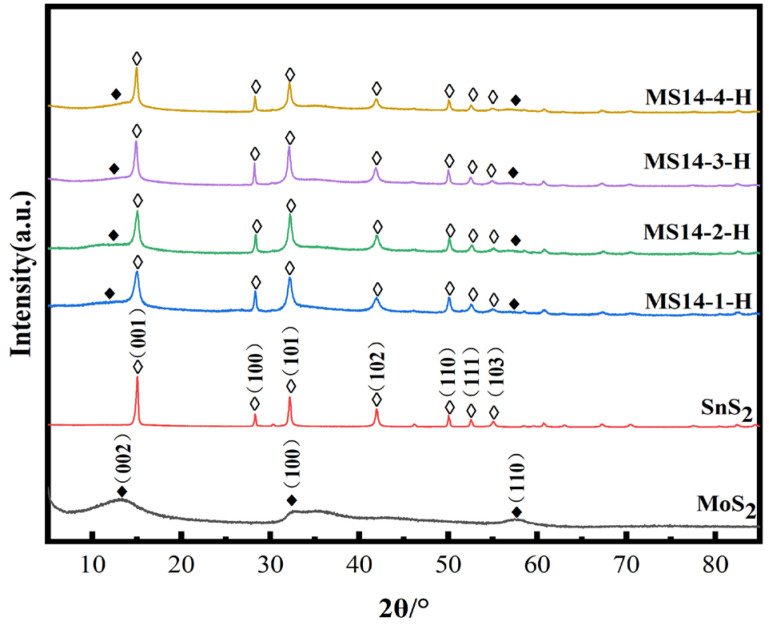
Powder X-ray diffraction patterns of MoS_2_/SnS_2_ composite catalysts synthesized at different molybdenum–tin molar ratios.

Figure 6 represents the XRD patterns of the composite catalysts synthesized by varying the molybdenum–tin molar ratio in the reactants at a reaction temperature of 180 °C for 14 h. It can be seen from the figures that the characteristic peaks at 2θ equal to 14.9°, 28.2°, 32.1°, 41.8°, 49.9°, 52.4° and 54.9° correspond to the (001), (100), (101), (102), (110), (111) and (103) crystal planes of SnS_2_ in the composite catalysts of different molybdenum–tin molar ratios, respectively [45]. In addition, no excess spurious peaks were found in the XRD patterns, indicating that the MoS_2_/SnS_2_ composite catalysts were successfully synthesized.

When increasing proportion of Mo in the reactants, the characteristic peaks of both MoS_2_ and SnS_2_ gradually broadened, especially the (100), (101) and (102) crystal planes in SnS_2_.

#### 3.2.2. Morphology Analysis

**Figure 7:**
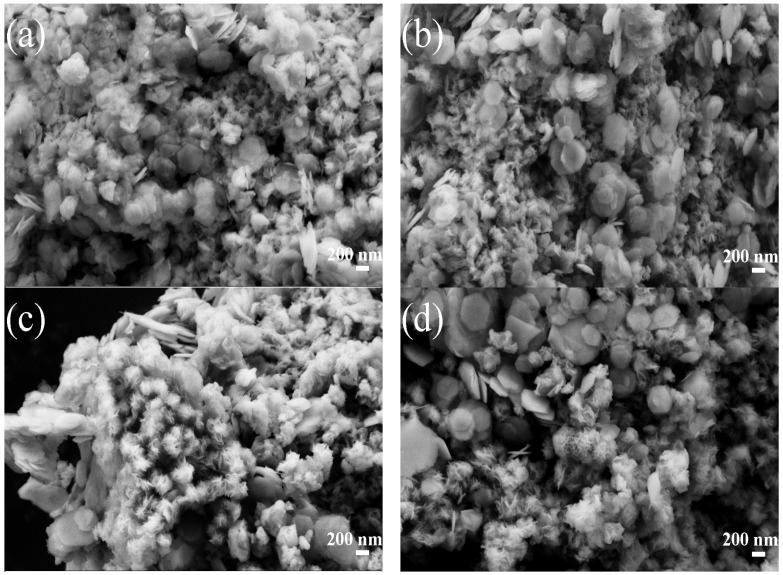
SEM diagrams of composite catalysts. (**a**) MS14-1-H. (**b**) MS14-2-H. (**c**) MS14-3-H. (**d**) MS14-4-H.

SEM images of the samples MS14-1-H, MS14-2-H, MS14-3-H and MS14-4-H are shown in Figure 7. From the figures, MoS_2_ particles are shown as having flower-like morphology composed of layered nanosheets. Increasing the proportion of Mo leads to more serious MoS_2_ agglomeration and the spherical flower particles of MoS_2_ become more regular. The growth of SnS_2_ nanosheets on the surface of the flower-like MoS_2_ morphology exhibits hexagonal morphology and the SnS_2_ nanosheets have a size of about 400 nm [1]. With an increase in the MoS_2_ fraction, the main change in the morphology is that the SnS_2_ nanosheets are not uniformly distributed on the MoS_2_ surface, as shown in Figure 7c,d. At lower Mo/Sn ratios, the scanning images of the catalyst changed more, and the MoS_2_ morphology was no longer a regular spherical flower shape but a nano-flake shape. In addition, the SnS_2_ nanosheets were closely distributed on the MoS_2_ surface, as shown in Figure 7a,b. The close distribution of the SnS_2_ nanosheets on the MoS_2_ surface contributes to the close bonding of the two materials, which can effectively change the electronic structure of the catalyst and increase the photocatalytic active sites.

**Figure 8:**
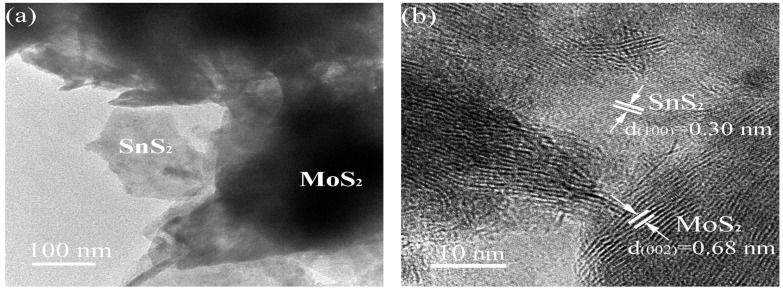
TEM images of MS14-2-H: (**a**) TEM image; (**b**) high-resolution TEM image.

Figure 8a shows TEM images of the prepared MS14-2-H catalyst, which further shows that the flake SnS_2_ grows on the surface of MoS_2_ with a small size. Lattice stripes with distances of 0.68 nm and 0.30 nm are shown in Figure 8b, which could correspond to the (002) plane of MoS_2_ and the (100) plane of SnS_2_, respectively [49]. These images objectively further explain the coexistence of SnS_2_ and MoS_2_ in the MS14-2-H composite catalyst. In addition to this, the lattice stripe in the (002) plane of MoS_2_ is larger than the standard spacing value (0.62 nm) according to the Bragg equation:

2dsinθ = nλ(2)

where d is the lattice spacing, θ is the angle between the incident ray, the reflection line and the reflected crystal plane, λ is the wavelength and n is the number of reflection levels. It is known that under specific conditions, the lattice spacing d is inversely proportional to the angle θ, i.e., at this time, the lattice spacing of the (002) plane of MoS_2_ is large and the corresponding diffraction peak angle becomes small, which is similar to the diffraction peak of the (002) corresponding to the MoS_2_ phase of the composite catalyst in the XRD analysis shifting to 10.9°.

#### 3.2.3. BET Measurements

Table 2 shows the results of the specific surface area tests of the MoS_2_/SnS_2_ composite catalysts with different molybdenum–tin molar ratios. From the data in the table, it can be observed that the specific surface area of the composite catalysts shows a trend of increasing and then decreasing when increasing the molybdenum–tin molar ratio from 1:1 to 4:1. In addition, the average pore diameter of the catalyst was 2.30~2.34 nm and the total pore volume was 0.003 cm^3^/g.

The effect of molybdenum–tin molar ratio on the average pore diameter and total pore volume of the composite catalysts is almost negligible, and the effect on the catalyst performance is mainly due to the specific surface area, which is 17.10 m^2^/g for MS14-2-H, followed by 15.05 m^2^/g for MS14-1-H. Combined with the analysis of the SEM images of the composite catalysts, the reason for the large differences in the specific surface areas of the catalysts may be due to the size and shape of the particles of MoS_2_ and SnS_2_ and the difference in the shape of the particles.

#### 3.2.4. Electrochemical Impedance Measurement

**Figure 9:**
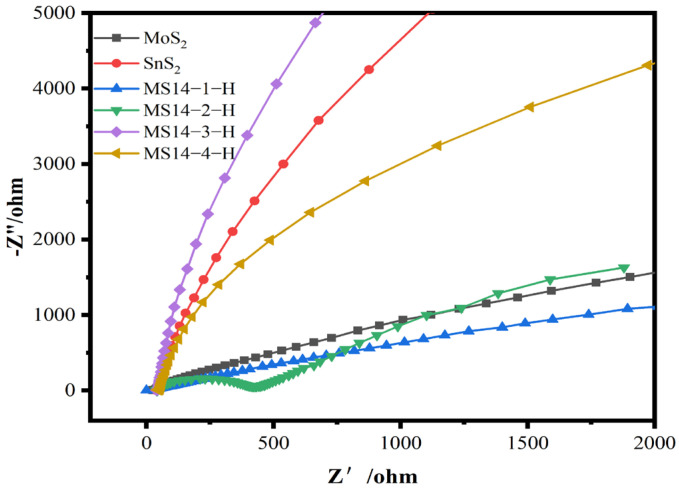
Electrochemical impedance plot of MoS_2_/SnS_2_ composite catalysts synthesized with different molybdenum–tin molar ratios.

The electrochemical impedance diagrams of the composite catalysts synthesized at different molybdenum–tin molar ratios are shown in Figure 9. According to the test results, the impedance radii of the MoS_2_/SnS_2_ composite catalysts showed a trend of decreasing and then increasing as the molybdenum–tin molar ratio increased; the MS14-2-H composite catalyst having the smallest impedance radius. Since the radius of the semicircle in the high-frequency region is positively correlated with the charge transfer resistance in the electrochemical impedance diagram, the sample MS14-2-H has the smallest charge transfer resistance [48].

#### 3.2.5. Structural Composition Analysis of Materials

**Figure 10:**
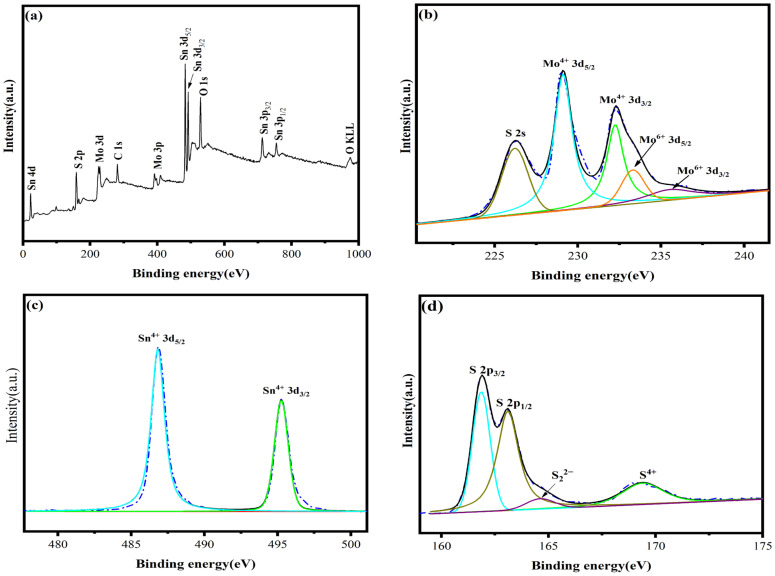
XPS diagram of catalyst MS14-2-H: (**a**) survey spectra; (**b**) Mo 3d; (**c**) Sn 3d; (**d**) S 2p.

Figure 10 shows the XPS diagram of the catalyst MS14-2-H. The elemental composition and elemental chemical valence of the sample catalyst can be identified by analyzing the XPS. From Figure 10a, it can be seen that the catalyst MS14-2-H contains C, O, S, Mo and Sn, and the sample contains all the elements of the target substance by the elemental full spectrum.

Figure 10b shows the XPS spectra of Mo 3d, in which five different characteristic peaks appear. It is known from the split peak fitting and literature review [50] that peaks at 229.1 eV and 232.2 eV correspond to Mo^4+^ 3d_5/2_ and Mo^4+^ 3d_3/2_, respectively, peaks at 233.3 eV and 235.5 eV correspond to Mo^6+^ 3d_5/2_ and Mo^6+^ 3d_3/2_, respectively, and the peak at 225.8 eV corresponds to S^2−^ 2s. The Mo^4+^ species belong to MoS_2_ and Mo^6+^ signals and may be caused by slight oxidation in air. Only two characteristic peaks appear in Figure 10c, and a review of the literature shows that [51] signals at binding energies of 486.8 eV and 495.3 eV correspond to Sn^4+^ 3d_5/2_ and Sn^4+^ 3d_3/2_, respectively, whereas the Sn^4+^ species belong to SnS_2_. Signals of S^2−^ 2p_3/2_ and S^2−^ 2p_1/2_ from the composite catalyst present at binding energies equal to 161.9 eV and 163.1 eV [52]. Therefore, the analysis shows that the composition of the synthesized samples is consistent with the target MoS_2_/SnS_2_ catalyst.

#### 3.2.6. Photocatalytic Performance

**Figure 11:**
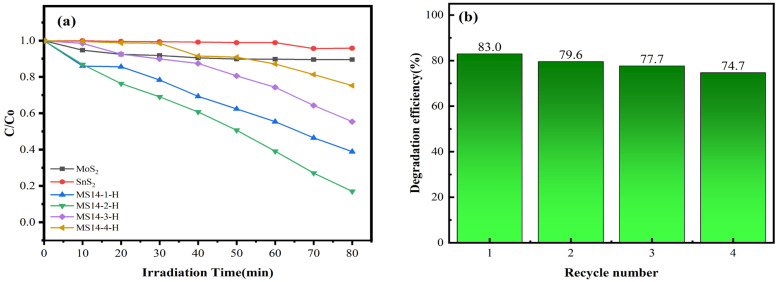
(**a**) Degradation rate diagram of MoS_2_/SnS_2_ composite catalysts synthesized with different molybdenum–tin molar ratios. (**b**) Cycling stability testing of the composite catalyst MS14-2-H.

Figure 11a shows the performance of the composite catalysts synthesized at different molybdenum–tin molar ratios in the degradation of methylene blue solutions. From the degradation data in the figure, the visible light degradation performances in methylene blue solution of the composite catalysts synthesized at different molybdenum–tin molar ratios are better than that of either pure MoS_2_ or SnS_2_, indicating that the photocatalytic performance can be effectively improved by using a composite of these two materials. In addition, after visible light irradiation for 80 min, the best catalytic performance was achieved for sample MS14-2-H, which had a degradation rate of 83%, whereas the degradation rate of sample MS14-3-H was 44.6% and that of sample MS14-4-H was 24.7%, indicating that the optimal molybdenum–tin ratio that can effectively improve the photocatalytic performance is 2:1 and that the effect on the photocatalytic performance is limited by only increasing the content of MoS_2_ in the composite catalyst. Attention should be paid to the reasonable distribution of the two components in the composite catalysts, which is consistent with the previous results of the XRD, SEM and EIS analyses.

Figure 11b shows the cycling stability test of the composite catalyst MS14-2-H. It can be seen from the figure that the catalytic degradation efficiency of the composite catalyst MS14-2-H in the MB solution decreased from 83.0% to 74.7% after four visible photocatalytic cycle tests and that the loss of photocatalytic activity was 8.3%. This indicates that the stability and repeatability of the composite catalyst MS14-2-H are good, whereas the loss of photocatalytic activity may be caused by the loss of photocatalysis during the cycle test [1].

### 3.3. Photocatalytic Mechanism

**Figure 12:**
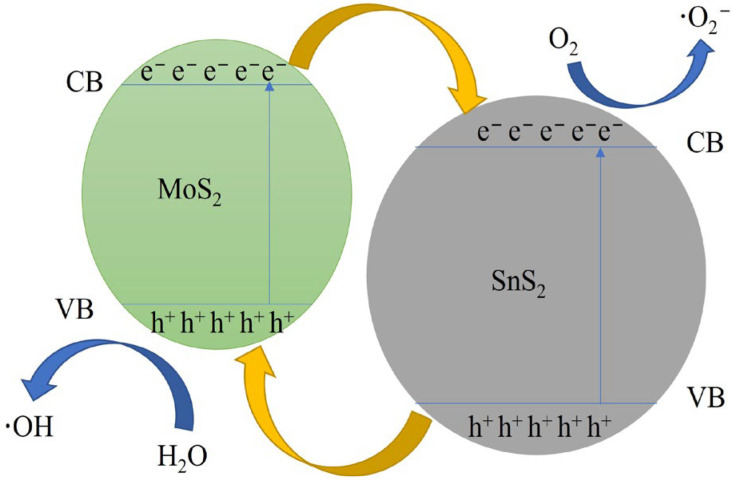
Possible photocatalytic mechanism for the degradation of methylene blue over a composite catalyst.

The photocatalytic mechanism of the composite catalyst is shown in Figure 12. MoS_2_ is a p-type semiconductor material with a narrow band structure (e.g., =1.85 eV), whereas SnS_2_ is an n-type semiconductor material with a forbidden band width of 2.08 eV [48]. Because the two semiconductors have opposite conductivity types, the electrons and holes of these two semiconductor materials are transferred when they are in close contact to form a heterojunction until the Fermi energy levels of the two semiconductor materials are equal, at which point the p–n heterojunction is in thermal equilibrium and a stable built-in electric field is formed.

In the mechanism diagram of the composite catalyst, the CB and VB of MoS_2_ are higher than that of SnS_2_, the energy band structures of both are staggered and the heterogeneous structure of the composite catalyst is of type II. When irradiated by visible light, a large number of photogenerated electrons accumulate in the conduction band and a large number of photogenerated holes accumulate in the valence band of both semiconductor materials. Under the effect of potential difference, electrons in the conduction band of MoS_2_ are transferred to the conduction band of SnS_2_, whereas holes in the valence band of SnS_2_ are transferred to the valence band of MoS_2_. In this way, the electrons and holes can be separated to the maximum extent.

The photocatalytic degradation of MB by composite catalysts under visible light is mainly based on the chemical reaction of the photogenerated electron reduction transferred to the surface of the photocatalyst with dissolved oxygen, which produces strongly oxidizing superoxide radicals (·O^2−^), and the chemical reaction of the strongly oxidizing holes transferred to the surface of the photocatalyst with hydroxyl radicals (OH^−^) in water and aqueous solutions, which produces hydroxyl radicals (·OH) [1]. The photocatalytic reaction process is as follows:

MoS_2_/SnS_2_ + hν → e^−^ + h^+^ (3)

e^−^ + O_2_ → ·O^2−^ (4)

h^+^ + H_2_O/OH^−^ → ·OH (5)

·O^2−^ + MB → CO_2_ + H_2_O (6)

·OH + MB → CO_2_ + H_2_O (7)

## 4. Conclusions

In summary, a novel MoS_2_/SnS_2_ heterostructure was successfully prepared by growing SnS_2_ nanosheets on MoS_2_ nanospheres by a facile multi-step hydrothermal method. Based on the measurements of the XRD, SEM, HRTEM and XPS analyses, the present composite sample was found to have high crystalline quality and excellent heterojunction formation. By constructing heterojunctions between the two sulfides, an improved photocatalytic performance was achieved, which greatly solved the problems of low visible light utilization and photogenerated charge recombination. Compared with pure MoS_2_ or SnS_2_, this easily accessible and simple composition photocatalyst shows higher photocatalytic activity and good photostability; these effects are attributed to the constructed heterostructure, better light trapping and rapid separation and migration of light-induced electron and hole pairs with the assistance of the MoS_2_ metal phase. The optimal MoS_2_/SnS_2_ photocatalyst (i.e., the one that achieved the best photocatalytic performance) had a degradation efficiency of 83.0% for MB solution, which was 8.3 times higher than the degradation with pure MoS_2_ and 16.6 times higher than the degradation with pure SnS_2_. The experimental results indicate that this construction of heterojunctions between semiconductors can effectively improve the photocatalytic ability of MoS_2_/SnS_2_ catalysts in terms of MB degradation.

## Figures and Tables

**Table 1:** BET analysis of composite catalysts for different reaction times.

Catalysts	BET Surface Area (m^2^/g)	Average Pore Width (nm)	Total Pore Volume (cm^3^/g)
MoS_2_	5.03	2.03	0.001
SnS_2_	9.00	2.02	0.003
MS12-2-H	13.07	2.32	0.003
MS14-2-H	17.10	2.34	0.003
MS16-2-H	12.05	2.32	0.003

**Table 2:** BET analysis of composite catalysts with different Mo–Sn molar ratios.

Catalysts	BET Surface Area (m^2^/g)	Average Pore Width (nm)	Total Pore Volume (cm^3^/g)
MoS_2_	5.03	2.03	0.001
SnS_2_	9.00	2.02	0.003
MS14-1-H	15.05	2.32	0.003
MS14-2-H	17.10	2.34	0.003
MS14-3-H	12.75	2.30	0.003
MS14-4-H	11.50	2.31	0.003

## Data Availability

The data presented in this study are available on request from the corresponding author.
